# Synthesis and Effect of Hierarchically Structured Ag-ZnO Hybrid on the Surface Antibacterial Activity of a Propylene-Based Elastomer Blends

**DOI:** 10.3390/ma11030363

**Published:** 2018-03-01

**Authors:** Pavel Bazant, Tomas Sedlacek, Ivo Kuritka, David Podlipny, Pavlina Holcapkova

**Affiliations:** Centre of Polymer Systems, Tomas Bata University in Zlin, Trida Tomase Bati 5678, 760 01 Zlin, Czech Republic; sedlacek@utb.cz (T.S.); kuritka@utb.cz (I.K.), podlipny@utb.cz (D.P.); holcapkova@utb.cz (P.H.)

**Keywords:** Ag-ZnO, thermoplastic elastomers, polypropylene, nanocomposites, hierarchical, antibacterial

## Abstract

In this study, a hybrid Ag-ZnO nanostructured micro-filler was synthesized by the drop technique for used in plastic and medical industry. Furthermore, new antibacterial polymer nanocomposites comprising particles of Ag-ZnO up to 5 wt % and a blend of a thermoplastic polyolefin elastomer (TPO) with polypropylene were prepared using twin screw micro-compounder. The morphology and crystalline-phase structure of the hybrid Ag-ZnO nanostructured microparticles obtained was characterized by scanning electron microscopy and powder X-ray diffractometry. The specific surface area of this filler was investigated by means of nitrogen sorption via the Brunauer-Emmet-Teller method. A scanning electron microscope was used to conduct a morphological study of the polymer nanocomposites. Mechanical and electrical testing showed no adverse effects on the function of the polymer nanocomposites either due to the filler utilized or the given processing conditions, in comparison with the neat polymer matrix. The surface antibacterial activity of the compounded polymer nanocomposites was assessed against *Escherichia coli* ATCC 8739 and *Staphylococcus aureus* ATCC 6538P, according to ISO 22196:2007 (E). All the materials at virtually every filler-loading level were seen to be efficient against both species of bacteria.

## 1. Introduction

Recent years have witnessed a growing interest in developing polymer nanocomposites containing hybrid nanoparticles [1]. Advances made in polymeric nanocomposites have ushered in a new generation of macromolecular materials with low densities and multifunctional properties [2]. The primary advantage of nanocomposites is the tiny amount of filler needed to fulfil the given requirements, which could be one or even two orders of magnitude less than required by conventional micro-fillers [3].

Such advanced polymer systems incorporate a hybrid metal semiconductor materials that has attracted particular attention. This is not only due to the fact that co-joined metal and semiconductor nanoparticles possess a large specific surface area and high fraction of surface atoms, but also because they feature a unique electronic band structure which results in certain chemical activity [4,5,6]. For instance, hybrid Ag-ZnO nanoparticles constitute a filler of such materials. Indeed, Ag-ZnO hybrid nanoparticles are of particular interest since they exhibit biological, and photocatalytic activity, low toxicity, and exert a synergetic antibacterial effect.

In addition, adding said nanoparticles into a polymer gives rise to new, hybrid systems with combined properties and even some synergies. This was successfully proved for polymer melt compounded materials having further utilisation in medical, agricultural and catalytic applications [7,8,9]. Some research studies aim to eliminate bacterial growth and pathogen formation on various surfaces of subjects, examples being electrical equipment, walls, tables, floors, cars, food packaging, public and interior spaces and hospital facilities [10,11,12,13,14,15,16].

Nevertheless, nanoparticles may potentially cause problems due to safety issues. The occupational health risks associated with manufacturing and utilizing nanomaterials are not yet clearly understood [17,18,19,20]. Moreover, nanoparticles are difficult to process into thermoplastic polymers by conventional methods, e.g., extrusion, melt-blow, compression or injection moulding, as the filler forms micron-sized cluster-agglomerates and possesses a non-homogenous distribution. Therefore, dispersion and distribution of the nanoparticles in the polymer matrix can be spatially varied in the volume of prepared plastic article. Moreover, the surface of the product, if rendered as a thin film, can show serious surface and optical defects. In addition, during the thermoplastic processing of nanoparticles, hot spots can occur in the material adhering to heating elements, along with burns, and a further disadvantage is a greater amount of non-processable waste [21,22,23,24,25].

One way to avoid the problems described above is to use nanostructured hierarchical microparticles. These three-dimensional materials can offer advantages arising from their large surface area, as building blocks are often nanoscale in extent. Nonetheless, primary nanocrystals have been known to assemble themselves into complex structures, such as hierarchical flowers or spheres, these ranging in size from mesoscale to microscale; this is a phenomenon driven by reduction in overall system energy during synthesis. Therefore, multiscale hierarchical nanostructured materials are promising due to (i) properties gained when their building blocks are nanoscale in size and (ii) composite processability arising when at meso to micro scale [26,27].

A variety of synthesis methods for preparing Ag-ZnO have been reported. However, most are limited to research purposes as a consequence of the need for high temperature and pressure, expensive equipment, toxic reagents and a long reaction time [28,29,30,31]. In addition, many synthesis techniques utilize extremely dilute solutions as the resultant nanoparticles have to possess a specific structure [32,33,34,35]. These methods are not easily scalable to produce sufficient amounts of filler for typical commercial means of processing plastic, e.g., extrusion, melt-blow, compression and injection moulding.

In this paper, the authors offer up an original methodology for generating polymer nanocomposites by melt-mixing a pre-prepared filler, which involves adding Ag-ZnO hierarchical microparticles to the polymer matrix, in this case a blend of polypropylene and thermoplastic polyolefin elastomers (TPOs). In less than a decade, TPOs, defined as materials combining small enough semi-crystalline domains connected via amorphous elastomeric regions and renowned for their adjustable rubber-like characteristics, have emerged as a core material processed by standard thermoplastic processing equipment in automotive interiors and exteriors, buildings, wires and cables, film applications, medical devices, adhesives, footwear, foams, and other extruded and moulded goods [36,37,38].

Proposed methodology of compounding secured not only disagglomeration of processed micropartices into their building nano-blocks by shear forces during mixing but also ensured good distribution and dispersion of the resultant nanoparticles. Preparing the Ag-ZnO hierarchical particles merely calls for application of a facile hydrothermal method, even facilitating large-scale production of the same. It will be viable if the chemicals in the filtrate are recovered and reused, namely silver. Said polymer nanocomposites demonstrate excellent antibacterial properties against Staphylococcus aureus and Escherichia coli, as well as boasting unwavering mechanical and electrical properties.

## 2. Materials and Methods

### 2.1. Materials

Silver nitrate AgNO_3_ (>99.5% purity) and Zinc nitrate hexahydrate (ZnNO_3_)_2_·6H_2_O (>99% purity) were delivered by Penta (Praha, Czech Republic), while ammonium carbonate (NH4)_2_·CO_3_ (>99% purity) was supplied by Sigma-Aldrich (Praha, Czech Republic). Demineralized water was used throughout the experiments. Thermoplastic polyolefin elastomer of type Versify 3401 (Dow Europe GmbH; Rheinmünster, Germany) and polypropylene random copolymer TOTAL PPR 6298 S01 (Total Petrochemicals & Refining S.A./N.V., Bruxelles, Belgium) were utilized as the polymer matrix or blends, respectively.

### 2.2. Synthesis of Ag-ZnO

Firstly, a solution of 0.1 mol (ZnNO_3_)_2_·6H_2_O was mixed together with a solution of 0.01 mol AgNO_3_; the total volume of water used to dissolve both salts was 100 mL; the amount of 0.125 mol (NH_4_)_2_·CO_3_ was dissolved in 125 mL of water separately. The solution of ammonium carbonate was slowly added, during a period of 10 min, into the solution comprising (ZnNO_3_)_2_·6H_2_O and AgNO_3_, which was stirred vigorously throughout. The end product was washed by filtration and the powder obtained then dried at 100 °C for 8 h, then the intermediate was annealed at 450 °C for 2 h to obtain product.

### 2.3. Preparation of Nanocomposites

Pellets of the polypropylene (PP) and thermoplastic polyolefin elastomer (TPO) were premixed with the prepared filler and then the material was compounded by a co-rotating conical twin screw extruder Micro-Compounder Xplore MC15 (DSM Xplore Instruments BV, Sittard, The Netherlands). Melt treatment of a mixture at the speed of 50 rpm for 4 min at temperature set to 200 °C for the barrel, the die and the circular loop guaranteed achieving of a constant torque, thus demonstrating that stabilization had occurred, thereby indicating that the filler had been homogeneously mixed into the matrix. Afterwards, extruded strands were cooled in air and cut to the pellets. The filler concentrations 1, 3, and 5 wt % were chosen by the authors; hence, hereinafter the given filler containing compound is referred to as Ag-ZnO1, Ag-ZnO3 or Ag-ZnO5.

Consequently, sheets of the polymer nanocomposites, measuring 1 mm and 4 mm in thickness, were produced by the hot press method (Fontijne LabEcon 300, Barendrecht, The Netherlands); this involved preheating at 200 °C for 2 min, followed by compression for 4 min and subsequent cooling under pressure of 200 kPa. A pure PP and a PP/TPO blends without any filler were prepared in exactly the same way to obtain reference samples. The sheets obtained were applied as testing samples for evaluating mechanical properties, electrical resistivity and antibacterial activity.

The aforementioned pure PP and PP/TPO blends were processed with various PP/TPO weight contents, namely 100/0, 75/25 and 50/50 *w*/*w*, respectively. Hereinafter, these blends are referred to as PP100, PP75, PP50.

### 2.4. Structural Characterization

The crystalline structure of the powder obtained was characterized by X-ray diffraction (XRD), on a Rigaku Miniflex 600 X-ray diffractometer (Rigaku Europe SE, Neu-Isenburg, Germany) operated at 40 kV and 15 mA. Nickel-filtered Co Kα1 radiation (λ = 1.78892 Å) was applied over a 25–90° angular region, with the step size and rate set to 0.02° and 10°/min, respectively. The crystallite size (*d_diffr_*) of the samples was estimated using the Scherrer Equation (1):

*d_diffr_* = (0.9λ)/(βcosθ) (1)

This involved gauging the line-broadening (β, full width at half-maximum, corrected by the response of the instrument) of the main peak intensity for zinc oxide (101) and (111) for silver, where λ is the wavelength for Co Kα1 radiation and 2θ is the Bragg angle. Phase composition was evaluated via Rigaku Miniflex 600 software, utilizing the normalized RIR method. The RIR is the ratio between the integrated intensities of the peak of interest and that of a known standard.

The micrographs of the prepared powder and nanocomposites were taken on scanning electron microscopes, a Phenom Pro (Phenom-World, Eindhoven, The Netherlands) unit for preliminary and overall observations, and a Nova NanoSEM 650 (FEI, Brno, Czech Republic) unit equipped with a backscattered electron (BSE) detector for detailed imaging. Foregoing coating of examined samples comprising a thin layer of gold/palladium had been applied by a SC 7640 sputter coater (Quorum Technologies, Lewes, UK).

The specific surface area *A_BET_* was determined via multipoint Brunauer-Emmet-Teller (BET) analysis of the nitrogen adsorption/desorption isotherms at 77 K, recorded on Belsorp-mini II (BEL Japan Inc., Osaka, Japan) apparatus. Grain size is expressed as the mean diameter *d_BET_*, according to Equation (2) [39]:

*d_BET_* = 6/(*ρ_s_*·*A_BET_*) (2)

where *ρ_s_* is the density of adsorbent material. The equation is based on spherical approximation of the compact shape of grains.

The method of Barrett, Joyner, and Halenda (BJH) was employed for calculating pore size distributions from experimental isotherms using the Kelvin model of pore filling. Pore size distribution is calculated from desorption isotherm.

### 2.5. Investigation of Mechanical Properties

Tensile and three point flexural tests were carried out on a Testometric universal-testing machine of type M 350-5CT (Testometric Co. Ltd., Rochdale, UK), equipped with a load cell of 300 kN. The tensile properties of the polymer nanocomposites were investigated using a crosshead speed of 50 mm/min and the length of the gauge equalled 50 mm. The dumb-bell-shaped specimens of Type 2 (specified by ISO 527) of the thickness 1 mm were cut out from prepared sheets by the means of punching press. In total, six specimens of each material were tested.

Five individual samples of dimensions 80 mm × 10 mm × 4 mm, according to the ISO 178 standard [40], for all the compositions were milled out from the 4 mm pressed sheets using CNC milling machine Charly4U (Mecanumeric Co., Motta di Livenza, Italy) in order to define both the flexural strength and modulus of the same. A span of 64 mm was applied, pertaining to a span to depth ratio of 16:1. The samples were positioned in the middle of the supports, and the device was operated at a speed of 1.0 mm/min. under ambient temperature conditions.

### 2.6. Investigation of Electrical Properties

The surface and volume resistivity of the prepared nanocomposite films (according to the ASTM D257 standard [41]) was evaluated by means of a Keithley 8009 Resistivity Test Fixture, on a Keithley 6517A Electrometer/High Resistance Meter (Keithley Instruments Inc., Cleveland, OH, USA). Surface and volume resistivity was obtained under a DC voltage of 40 V after a bias time of 60 s. The testing specimens in the shape of discs of a diameter 65 mm and thickness 1 mm were cut out from the pressed sheets.

The electrical breakdown strengths of the polymer nanocomposites were gauged using a GLP1-g High-Voltage Tester (Schleich Co., Hemer, Germany), with measurements being taken eight times for each specimen for reasons of accuracy. A DC voltage was slowly applied to the sample(s) at a rate of approximately 0.1 kV per second, and each voltage was maintained for 10 s in order to evaluate the breakdown strength of said sample. The upper limit of current drawn during the experiment equalled 6 mA. All the tests were carried out at room temperature.

### 2.7. Evaluation of Antibacterial Activity

The surface antibacterial activity of the prepared compounds was assessed in vitro against *E. coli* ATCC 8739 and *S. aureus* ATCC 6538P, these comprising the representative strains of gram-negative and gram-positive bacteria, respectively; this was conducted according to ISO 22196:2007 (E) [42] “Plastics—measurement of antibacterial activity on plastics surfaces”. The dimensions of the sample, square test pieces of sheet were 25 mm × 25 mm × 1 mm. In light of previous experience with the tests, modification to the original protocol was made that was adherent with ISO 22196:2007 (E) [42], the purpose being to reduce the risk of false results. The number of colonies grown from recovered cells was estimated after 48 h of cultivation (instead of 24 h as required by the original standard procedure) to ensure that all the colonies had developed to form countable sizes. Hence, the overall duration of the test after inoculation was 48 h at 35 °C [5,19]. An incubator, a HERAcell 150i model (Thermo Scientific, Waltham, MA, USA), was applied in this part of the work. Antibacterial activity, delineated as R, was calculated by Equation (3):

R = (U_t_ − U_0_) − (A_t_ − U_0_) = U_t_ − A_t_ (3)

where: R is antibacterial activity; U_0_ is the average logarithm for the number of viable bacteria, in cells/cm^2^, recovered from untreated test specimens immediately after inoculation; U_t_ is the average logarithm for the number of viable bacteria, in cells/cm^2^, recovered from untreated test specimens after 48 h; and A_t_ is the average logarithm for the number of viable bacteria, in cells/cm^2^, recovered from treated test specimens after 48 h [5,19]. Indeed, in several cases, some additional colonies were found by this procedure, which avoided incorrect overestimation of antibacterial activity as caused merely by the slower growth of the colonies.

Converting from logarithmic (base 10) reduction R to the percentage of reduction (P) is possible through applying the following formula: P = 100 × (1 − 10^−^^R^). This means that the *R*-values (log_10_ scale) of 1, 2, 3, 4, 5 and 6 correspond to reductions in microbial load of 90%, 99%, 99.9%, 99.99%, 99.999% and 99.9999%, respectively.

## 3. Results and Discussion

### 3.1. Crystal Structure and BET Characterization of the Filler

**Figure 1:**
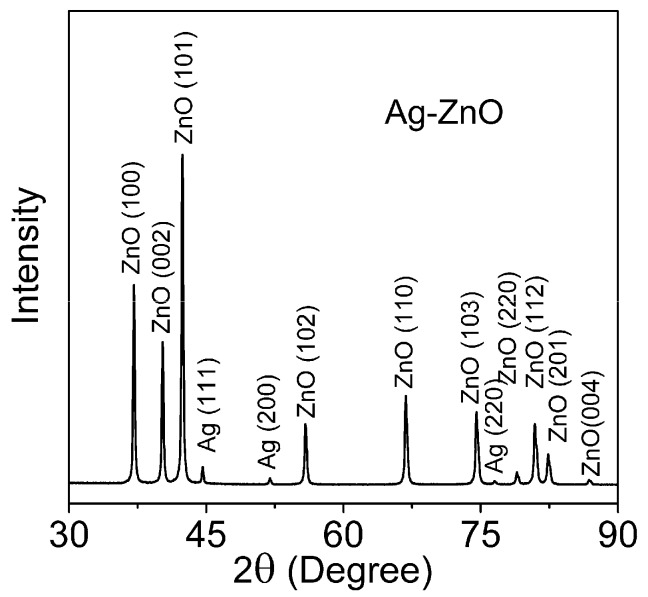
XRD pattern for the prepared Ag-ZnO hierarchical microparticles obtained by the synthetic method.

The powder XRD pattern for the prepared Ag-ZnO filler is shown in Figure 1. All diffraction peaks observed at 2θ = 37.06°, 40.19°, 42.35°, 55.80°, 66.78°, 74.49°, 78.97°, 80.94°, 82.38° and 86.86° are characteristic for the wurtzite ZnO structure (hexagonal phase, space group P63mc) and are in good agreement with the JCDD PDF-2 entry 01-079-0207. Diffraction peaks at 2θ = 44.60°, 51.99° and 76.53° correspond well with the fcc crystal structure of silver detailed in the JCDD PDF-2 entry 01-087-0720.

Quantitative analysis of the XRD pattern, performed via the reference intensity ratio (RIR) method (applying the Corundum standard [43]), showed that the content of silver in the Ag-ZnO sample was merely 1.3 wt %, with the remaining 98.7% constituting the wt % of ZnO. This confirms the presence of the target amount of metallic Ag on said ZnO. The fact that there is no shift in the peak position for the ZnO phase in the sample indicates that Ag^+^ ions either enter into the lattice of the ZnO or substitute a Zn site [44]. It is reasonable to expect, that metallic Ag particles are positioned on the surfaces or interfaces of the ZnO nanoparticles. Other Ag peaks were not observed in the XRD patterns, which is probably due to the small quantity of Ag nano-particles and their excellent dispersion.

The diffraction lines from the (101) and (111) planes in ZnO and Ag, respectively, as visible in the diffractogram images at 2θ angle 42.42° and 44.60°, were applied so as to deduce the diffracting area size *d_diffr_*. Findings pertaining to the structural and morphological parameters obtained by XRD are displayed in Table 1. Assuming that the grains are spherical in shape and of uniform size, average particle size can be obtained via Equation 2 with respect to grain size *d_BET_*. Thus, the analysis revealed the figure to be 64 nm. The average material density of Ag-ZnO was obtained from the material composition estimated by XRD assuming the tabular material density of silver (10.49 g/cm^3^) and ZnO (5.61 g/cm^3^). The specific surface area for a crystallite (*A_diffr_*) is easily calculated from the size of the crystallites (*d_diffr_*) and the material density of Ag-ZnO (5.64 g/cm^3^). According to analysis of sorption/desorption isotherm, the pore sizes of our sample are expected to be approximately 17 nm. Although pores may not be truly cylindrical, we refer to their sizes as diameters consistent with Kelvin model, thus making the measurement error to be acceptable; therefore, we used the simple BJH method.

Comparison can be made of results obtained from X-ray diffraction line-broadening analysis and those obtained from gas sorption BET analysis. The first and usually the most valuable information determined by BET concerns specific surface area *A_BET_*. As can be expected, *A_BET_* is lesser than the specific crystallite surface area. The X-ray diffraction characteristics are correctly obtained for coherently diffracting areas, i.e., pertaining to the size of the nanocrystalline domains, while gas sorption analysis examines the actual surface of the porous body accessible to N_2_ molecules; the latter analysis characterises the surface of grains. Consequently, the average diameter of grain is bigger than that of nanocrystals. Hence, *A_BET_* relates to the surfaces of grains that consist of one or more crystallites and to the amorphous phase, which should be present. Interface between congruent particles is not accessible to nitrogen adsorption becoming thus virtually invisible for BET measurement. It can be estimated from third powers of grain and nanocrystallite diameter that one grain comprises of approximately two nanocrystallites only. Such small packing level testifies for chain-like, weakly branched or twinning morphology of grains.

Higher activity of Ag-ZnO hierarchical particles with polymer matric against the microparticles due to relatively the large surface area, can be assumed, therefore some features of the composite have been improved.

### 3.2. Morphology of the Powder Filler

**Figure 2:**
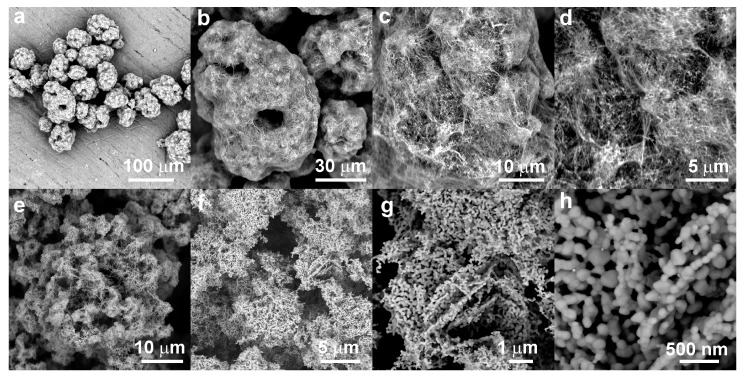
SEM microphotographs of the Ag-ZnO filler (**a**–**d**) before annealing and (**e**–**h**) after the annealing process.

Microphotographs of the prepared filler obtained by SEM are shown in Figure 2. Here, Figure 2a,d shows the intermediate. An overall image in Figure 2a shows large agglomerates possessing diameter up to 70 μm. They exhibit complicated hierarchical morphology. A large agglomerate comprises aggregates of the layered zinc hydroxide nitrate complex containing most likely nanoparticles of silver oxide, as can be expected to be a product of mild basic precipitation conditions in the first stage of synthesis. The apparent size of these primary agglomerates is about several micrometers and they are arranged into grape-like assemblies that seems to be bound and covered by a network of fibres. Their morphology resembles a shrub covered by web produced by Spindle Ermine (*Yponomeuta cagnagella*) moth caterpillars. The diameters of fibers measure up to 200 nm, while their lengths are equal to tens of microns (Figure 2b,d).

It can be expected that these fine fiber structures are created in later stages of synthesis. During annealing of the zinc complex, conversion is made to the nanostructured hybrid Ag-ZnO microparticles resembling shape of original intermediate agglomerates and keeping their hierarchical morphology. Thermal decomposition of zinc hydroxide nitrates yields spherical, connected microparticles of ZnO, which go on to form long chains and two-dimensional networks of polyhedral to rounded nanoparticles, as seen in Figure 2e,h. The ZnO phase obtain this morphology during annealing as a legacy of original layered morphology of zinc hydroxide nitrates by topotactic transition [25,45]. The diameters of the ZnO nanoparticles range up to 100 nm in size. Figure 2h details the nanoparticle building blocks. The size and morphology of particles revealed by the SEM is in good agreement with results of BET analysis.

### 3.3. Characterization of TPO Compounds

**Figure 3:**
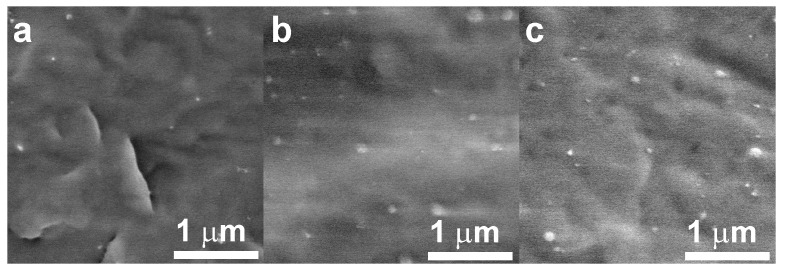
SEM microphotographs of fracture surfaces of polymer nanocomposites with Ag-ZnO particles: (**a**) PP100/Ag-ZnO5; (**b**) PP75/Ag-ZnO5; (**c**) PP50/Ag-ZnO5.

The morphology of the prepared polymer nanocomposites with 5 wt % of filler was analysed by SEM microscopy on the surfaces obtained by freeze fracturing in liquid nitrogen. Good dispersion and distribution of the filler in the polymer matrix is visible in Figure 3 recorded as representative images for typical sample fracture surface. As was demonstrated by SEM analysis, the filler is comprised from sparsely networked chains of nanoparticles that can be considered loosely bound. The interface between nanoparticles in long chains may be the weakest points and thus places where the cohesive forces of the synthesized ZnO microparticles can be overcome during compounding, a phenomenon which arises through high shear and elongation stresses, pertaining to reduction in the size of a component with sub-critical cohesive properties during compounding by mixing the molten phase. Therefore, large agglomerates of the filler were dispersed in the polymer matrix on individual particulates of the Ag-ZnO nanoparticles, said particulates measuring up to 100 nm in diameter and these single particulates dispersed in the polymer matrix are evident in Figure 3 for all compared nanocomposites. Separation of the silver nanoparticles from ZnO nanoparticles was neither confirmed nor excluded due to their very low concentration.

### 3.4. Mechanical Properties of the Polymer Nanocomposites

The authors chose to characterize the mechanical performance of the prepared nanocomposites according to their basic characteristics, i.e., yield stress, strain at break and flexural modulus, with adherence to ISO 527 [46] and ISO 178 [40]; the subsequent results are summarized in Table 2. Comparing the neat polymer matrix TPO and PP with all the prepared nanocomposites revealed that no adverse effects were exerted on the mechanical properties of the prepared material, either through using the fillers or the given weight percent of the same or through the processing conditions applied. Indeed, the mechanical properties e.g., flexural modulus, strain at break of the polymer nanocomposites and PP/TPO blends were only influenced by the amount of TPO material present dramatically. The yield stress and flexural modulus of the polymer nanocomposites slightly decreased alongside parallel increase in strain at break.

It cannot be expected, the mechanical properties will be dramatically influence by used spherical hierarchical microparticles of low-loading filler. On the other hand, the wettability and compatibility of hierarchical microfiller by thermoplastic polymer matrix plays important role for transport mechanical stress from polymer to filler.

### 3.5. Electrical Properties of the Nanocomposites

Zinc oxide is a well-known semiconductor with a broad range of resistivity, which not only depends on the morphology of the materials, but especially on the type and concentration of a dopant. The resistivity can be varied within the range 10^−4^ to 10^9^ Ω·cm by doping. This means that ZnO can successfully be prepared even in a conductive state, although a heavily doped material and special conditions are required [47]. In contrast, silver is an excellent metallic conductor with a resistivity of 1.59 × 10^−6^ Ω·cm [48]. Therefore, a combination of these materials could produce a hybrid with reasonably high electrical conductivity, while Ag-ZnO as a powder filler would cause conductivity, even under circumstances of low content of the filler in the polymer matrix, if the critical threshold value is exceeded [6].

Nevertheless, due to their chemical nature, most TPO compounds and PP are fine electrical insulating materials; they boast good dielectric strength and do not absorb moisture. To check the performance of prepared nanocomposites, electrical properties were tested with special attention paid to the electrical strength as a critical phenomenon related with the material endurance and failure.

Table 3 summarizes the electrical surface, volume resistivity and dielectric strength for all the neat and prepared materials. The surface resistivity of the neat PP and PP/TPO blends lies in the region 3.2 × 10^12^ to 1.1 × 10^13^ Ω/sq, while volume resistivity ranges 7.2 × 10^15^ to 9.8 × 10^15^ Ω·cm. The surface resistivity and volume of the neat PP and PP/TPO blend correspond to literature values [49], whereas the hybrid, nanostructured Ag-ZnO microparticles (compressed into disc-shaped pellets) exhibited the surface resistivity value 6.6 × 10^6^ Ω·cm, which resembles the figure for semi-conductors. The values observed for resistivity are almost identical for the polymer nanocomposites filled with Ag-ZnO and the blend matrixes of neat PP or PP/TPO. Hence, it is evident that the resistivity of the composite material is fully governed by the properties of the given matrix.

Although the filler is not conductive enough to establish conductivity due long-range connectivity of the particles at high concentration, it may affect the critical behaviour of the material under condition of electrical breakdown. The data on dielectric strength for all the samples are shown in Table 3. When comparing them, the results are obvious: the values for dielectric strength vary insignificantly for the PP/TPO blend. The polypropylene and pure blends reach dielectric strength at approximately 17 kV/mm. This is in contrast with the polymer nanocomposites, where a small yet pronounced trend can be observed. These exhibit lower values of only 13.6 to 15.5 kV/mm. The decrease in breakdown strength is attributed to the significant inhomogeneity of the local electric field, caused by differences between the polarizability of the dielectric polymer matrix and the dielectric microparticles of the filler.

### 3.6. Surface Antibacterial Activity

The relatively finite concentration of the filler utilized should not influence the mechanical or electrical properties of the neat PP or PP/TPO blend dramatically; indeed, it was observed. However, antibacterial activity has to be significantly evident if the filler can truly impart this desired property to prepared compounds even at a 1 wt % loading. Herein, the antibacterial activities of the polymer nanocomposite materials were tested according to the standard ISO 22196:2007 (E) [42] against *E. coli* and *S. aureus*.

The results are summarised in Table 4, where neat PP100, PP75 and PP50 comprise reference samples, giving the U_t_ value, while the *R* value indicates the surface antibacterial activity of all the prepared polymer nanocomposites. The earlier JIS Z 2801 standard [50], which preceded ISO 22196:2007 (E) [42], specified an *R*-value of 2.0 or more as demonstration of antibacterial activity. Therefore, most of the prepared polymer nanocomposite materials can be categorized as exhibiting antibacterial activity if they show values exceeding 2.0 on their surface; this categorization is brought about by compounding them with fillers under testing This critical value of 2.0 is adequate for hygienic and similar applications. Nonetheless, a value of 6.0, i.e., a 99.9999% reduction in cell count against controls, is considered applicable for advanced medical uses of plastics [26].

All the prepared polymer nanocomposites containing 5 wt % of nanostructured Ag-ZnO filler showed surface antibacterial activity against *E. coli* of greater than 6.2. The surface antibacterial activity of said polymer nanocomposites against *S. aureus* exceeds 2.5, although only the PP100/Ag-ZnO5 sample demonstrated an *R*-value that reached 4.8. With respect to the peculiarity of *S. aureus* inhibition, this result can also be evaluated as excellent, as the lower sensitivity of *S. aureus* to antibacterial agents is generally recognized [6,9,20,26,51]. The antibacterial activity against both bacteria is lower for 3 wt % content, but it is still sufficient for utilization in hygienic applications. It can be seen from Table 3, that the higher concentration of TPO is present in the compound the lower *R*-value is experienced. This effect is more pronounced for *S. aureus* and for lower concentrations of the filler.

The antimicrobial effects of materials coming from the mixture of an antimicrobial agent and a non-active polymer are similar to some extent as the mechanism of the agent itself. The possible mechanisms of killing microorganisms by Ag-ZnO filler were partly discussed elsewhere [6,8,20]. The effect of silver ions may be explained as follows: (1) uptake of free silver ions followed by disruption of ATP (Adenosine triphosphate) production and DNA replication, (2) silver nanoparticle and silver ion generation of reactive oxygen species (ROS), and (3) silver nanoparticle direct damage to cell membranes [52,53,54,55]. Similarly, the release of Zn^2+^ cations or its complex forms is one of the proposed mechanisms of ZnO′s activity [56] while the generation of reactive oxygen species (ROS) is another relevant explanation [57], but it requires illumination of the material by light with at least some portion of energy transferred by photons in the ultraviolet A (UVA) region [26,58]. Since the particles are embedded in the polymer matrix, it can be expected that mechanical detachment of the nanoparticles from the composite surface and their attachment to the surface of bacterial cells is not the principal mechanism imparting antibacterial property to the material. Previously reported synergy between silver and zinc oxide nanoparticles is based on direct contact and processes at the interface between the metallic particle and its semiconductor counterpart which may enhance both release of ions as well as ROS generation but not the mechanical particle action [8,20,51].

The presence of filler particles in the surface-region of the composites seems to be the first approach to understand observed trends in Table 4 [3,59]. Such direct mechanism would explain dependence of antibacterial activity on the filler concentration. However, a strong effect of used polymer matrix is clearly manifested too. Without any doubt, PP can be assessed as much efficient matrix than TPO. Embedding of particles into polymer matrix in both surface and near subsurface-region invokes a three step mechanism. First, it requires diffusion of water into the matrix, then corrosion of the filler particles resulting into the release of ions or ROS generation and finally, replenishment of active species on the polymer composite surface by diffusion [3,52].

According to the summary of results in Table 4, *E. coli* was more responsive to the effect of used filler than *S. aureus*. It can be explained by the difference in the cell wall structure. The thick peptidoglycan cell wall of Gram-positive bacteria protects its cell from silver penetration while Gramm-negative bacteria lack this protection [60,61]. Similar effect of the cell wall may apply for the case of Zn^2+^ ions as well as for ROS action [56,57,62].

The synergy effect of metal semiconductor hybrid Ag-ZnO antibacterial filler was demonstrated in our previous work although on material prepared by other synthetic routes [9,20,51]. However, increase of antibacterial efficiency towards *S. aureus* remains still a highly challenging issue which can be further influenced by the effect of polymer matrix choice as discussed above.

## 4. Conclusions

This study has detailed a method with real potential for preparing an antibacterial polymer nanocomposite. A conventional synthesis technique is applied that is simple, and information is given on how to avoid issues when processing the nanomaterials into the polymer matrix.

This filler boasts excellent homogenous distribution, dispersion and adhesion to selected representatives of polypropylene and thermoplastic elastomer and their blends. Furthermore, it can support the antibacterial performance of the polymer nanocomposite. The surface antibacterial activity observed herein of the prepared materials is assessed as excellent against *E. coli* and very high against *S. aureus*; hence they compare favourably against other materials available currently. It seems, that addition of TPO into polymer blend decreases the antibacterial activity of the nanocomposite keeping filler concentration constant. On the other hand, the mechanical and other properties of PP can be modified significantly by addition of TPOs in small concentrations only, as can be seen from relatively small effect of doubling the TPO concentration in the compound from 25 to 50 wt %.

Moreover, the mechanical and electrical properties of the polymer resin and blends utilized are not affected by adding a small amount of filler. Indeed, the prepared polymer nanocomposites possess the same resistivity as the neat matrix while dielectric strength is lowered only a little.

These facts testify to the fact that antibacterial polymer systems comprising Ag-ZnO nanostructured microparticles could potentially be employed as additives in plastic medical devices, in addition to finding uses in industries that require antibacterial action by a material, e.g., sanitary, hygienic or other interior applications.

## Figures and Tables

**Table 1:** Summary of XRD and BET analyses of Ag-ZnO. SSA stands for Specific surface area.

BET Method			XRD Method			
SSA, *A_BET_* (m^2^·g^−1^)	Grain Size, *d_BETm_* (nm)	Pore Size, (BJH) (nm)	Phase	Crystallite Size, *d_diffr_* (nm)	SSA for Pure Phase, *A_diffr_* (m^2^·g^−1^)	Contribution of Phases to SSA for Ag-ZnO (m^2^·g^−1^)
16.6	64	17	Ag	51	11.2	0.15
			ZnO	49	21.8	21.52
			Total SSA of Ag-ZnO, *A_diffr_* Ag-ZnO			21.7

**Table 2:** Mechanical properties of the compounds.

Samples	Yield Stress (MPa)	Strain at Break (%)	Flexural Modulus (MPa)
	Mean ± SD ^a^	Mean ± SD ^a^	Mean ± SD ^a^
Neat PP100	30.9 ± 2.2	14.5 ± 1.3	850 ± 50
PP100/Ag-ZnO1	34.2 ± 2.5	12.3 ± 1.4	840 ± 40
PP100/Ag-ZnO3	32 ± 3	13.6 ± 1.3	840 ± 40
PP100/Ag-ZnO5	30 ± 5	17.4 ± 2.6	830 ± 40
Neat PP75	18.8 ± 1.3	1120 ± 120	183 ± 15
PP75/Ag-ZnO1	18.6 ± 1.4	1100 ± 110	180 ± 19
PP75/Ag-ZnO3	18.5 ± 1.2	1035 ± 150	178 ± 17
PP75/Ag-ZnO5	18.5 ± 1.6	1020 ± 130	181 ± 20
Neat PP50	15.5 ± 0.9	1300 ± 130	34 ± 7
PP50/Ag-ZnO1	15.2 ± 1.3	1280 ± 150	32 ± 10
PP50/Ag-ZnO3	14.8 ± 0.7	1260 ± 130	37 ± 7
PP50/Ag-ZnO5	14.5 ± 1.1	1250 ± 170	38 ± 8

^a^ Standard deviation.

**Table 3:** Electrical properties of the compounds.

Sample	Resistivity, R		Dielectric Strength
	Surface Resistivity (Ω/sq)	Volume Resistivity (Ω·cm)	Mean ± SD ^a^ (kV/mm)
Ag-ZnO filler	-	6.6 × 10^6^	-
Neat PP100	1.1 × 10^13^	9.8 × 10^15^	17.4 ± 0.5
PP100/Ag-ZnO1	3.6 × 10^12^	5.5 × 10^15^	15.6 ± 1.4
PP100/Ag-ZnO3	3.0 × 10^12^	4.1 × 10^15^	14.8 ± 1.2
PP100/Ag-ZnO5	3.5 × 10^12^	4.2 × 10^15^	13.6 ± 0.6
Neat PP75	7.5 × 10^12^	8.7 × 10^15^	16.8 ± 0.7
PP75/Ag-ZnO1	3.2 × 10^12^	4.8 × 10^15^	15.5 ± 1.2
PP75/Ag-ZnO3	4.0 × 10^12^	5.5 × 10^15^	15.2 ± 1.3
PP75/Ag-ZnO5	3.7 × 10^12^	6.1 × 10^15^	14.1 ± 0.6
Neat PP50	3.2 × 10^12^	7.2 × 10^15^	17.2 ± 1.1
PP50/Ag-ZnO1	2.7 × 10^12^	5.5 × 10^15^	14.4 ± 1.2
PP50/Ag-ZnO3	3.0 × 10^12^	4.0 × 10^15^	15.5 ± 2.6
PP50/Ag-ZnO5	3.2 × 10^12^	3.9 × 10^15^	14.6 ± 1.4

^a^ Standard deviation.

**Table 4:** Evaluation of surface antibacterial activity of Ag-ZnO polymer nanocomposites according to ISO 22196:2007 (E) [42].

Sample	*R*-Value for *S. aureus* (-)	Efficiency against *S. aureus* (%)	*R*-Value for *E. coli* (-)	Efficiency against *E. coli* (%)
Neat PP100	U_t_ = 5.4		U_t_ = 6.2	
PP100/Ag-ZnO1	2.2	99.3690	4.2	99.9937
PP100/Ag-ZnO3	4.6	99.9975	5.5	99.9997
PP100/Ag-ZnO5	4.8	99.9984	>6.2	99.9999
Neat PP75	U_t_ = 5.7		U_t_ = 6.3	
PP75/Ag-ZnO1	0.7	80.0474	2.5	99.6838
PP75/Ag-ZnO3	2.2	99.3690	5.5	99.9997
PP75/Ag-ZnO5	2.8	99.8415	>6.3	99.9999
Neat PP50	U_t_ = 5.9		U_t_ = 6.3	
PP50/Ag-ZnO1	0.5	68.3772	1.5	96.8377
PP50/Ag-ZnO3	1.8	98.4151	2.5	99.6838
PP50/Ag-ZnO5	2.5	99.6838	>6.3	99.9999
